# Sex-Specific Dysregulation of Placental Lipid Metabolism in Preeclampsia

**DOI:** 10.26502/ogr0159

**Published:** 2024-07-23

**Authors:** Jay S. Mishra, Hanjie Zhao, Jing Zheng, Sathish Kumar

**Affiliations:** 1 Department of Comparative Biosciences, School of Veterinary Medicine, University of Wisconsin, Madison, Wisconsin, United States of America; 2 Department of Obstetrics and Gynecology, School of Medicine and Public Health, University of Wisconsin, Madison, Wisconsin, United States of America

**Keywords:** Preeclampsia, placental lipid metabolism, fatty acid oxidation, fatty acid esterification, fatty acid transport, fetal sex, sexual dimorphism, mitochondria, peroxisome, gene expression

## Abstract

**Background:**

Preeclampsia (PE) is a hypertensive disorder of pregnancy associated with adverse maternal and fetal outcomes. While placental dysfunction is implicated in PE pathogenesis, the impact of PE on placental lipid metabolism and its potential sexual dimorphism remains poorly understood.

**Methods:**

We conducted a comprehensive analysis of term placentas from PE and normotensive pregnancies with male and female fetuses. Lipid profiles were quantified using mass spectrometry, and mRNA expression of genes involved in fatty acid oxidation, esterification, and transport was assessed using qPCR.

**Results:**

Placentas from PE pregnancies exhibited elevated lipid levels, with male placentas showing a more pronounced increase in triacylglycerols, cholesteryl esters, and free cholesterol compared to female placentas. Gene expression analysis revealed sexually dimorphic alterations, with male PE placentas exhibiting upregulation of genes involved in fatty acid uptake, oxidation, and esterification, while female PE placentas showed a more complex response with both upregulation and downregulation of certain genes. Notably, peroxisomal fatty acid oxidation was upregulated in male PE placentas but suppressed in female PE placentas.

**Conclusions:**

Our findings reveal sexually dimorphic alterations in placental lipid metabolism in PE, suggesting that male placentas may be more vulnerable to lipotoxicity. These insights may have implications for understanding the pathogenesis of PE and developing sex-specific interventions to improve maternal and fetal outcomes.

## Introduction

Preeclampsia (PE), a hypertensive disorder of pregnancy affecting 5-7% of American women, poses significant risks to both maternal and fetal health [1]. This disorder not only poses immediate health risks but also predisposes the offspring to a higher likelihood of neurological and cardiovascular diseases in adulthood [2]. An impaired placental function may contribute to these adverse outcomes by disrupting the supply of nutrients, such as essential fatty acids, to the fetus [3, 4]. The transfer of fatty acids from the maternal to the fetal side is a vital process [5], mediated by placental plasma membrane-associated fatty acid transport proteins (FATP) and intracellular fatty acid-binding proteins (FABPs) expressed in trophoblasts [6, 7]. PE placentas exhibit lipid accumulation, inflammation, and oxidative stress [8-10], yet fatty acid uptake is often unchanged or reduced [11-14]. This lipotoxic state may arise from altered placental fatty acid metabolism, such as increased esterification or decreased β-oxidation and transplacental transport. Gene expression analysis of PE placentas reveals downregulation of genes involved in lipid transport (FATP 1 and 4) and fatty acid elongation (fatty acid desaturase 1), while the expression of fatty acid desaturase 2 and FABP3 remains unaffected [15]. These changes in placental fatty acid metabolism may influence fetal fatty acid delivery, impacting fetal growth, fat storage, and development [4, 16].

Comparative analyses reveal a significant decrease in placental mitochondrial function and/or abundance in PE pregnancies compared to controls [17-19]. This observation, coupled with the established role of mitochondria in fatty acid oxidation [20], suggests a potential downregulation of placental β-oxidation in the context of PE. For β-oxidation to occur, long-chain fatty acids (more than 14 carbons) must first be conjugated to carnitine for import into the inner mitochondrial membrane. This process is regulated by the activity of carnitine palmitoyltransferase (CPT)1, a mitochondrial enzyme responsible for the synthesis of acylcarnitines, a key regulator of β-oxidation rates [21, 22]. While carnitine is primarily obtained through dietary intake, it can also be synthesized endogenously in the kidneys, liver, brain, and possibly in the placenta [23-25]. Fetal carnitine is thought to be derived from maternal sources via transplacental transfer [26], facilitated by the OCTN2 transporter located in the syncytiotrophoblast plasma membrane [27]. In conditions of lipid excess, peroxisomes can contribute to fatty acid oxidation by shortening long-chain fatty acids, bypassing the CPT1 requirement for mitochondrial entry [28]. Peroxisome proliferation is induced by elevated lipid levels [29], suggesting a role for these organelles in PE and hyperlipidemic states, although their activity in PE placentas remains unclear.

Emerging evidence suggests sex-specific fetal responses to adverse intrauterine conditions result in differential susceptibility to metabolic diseases later in life [30]. Furthermore, placental function and response to maternal factors may vary depending on fetal sex. Rodent models have demonstrated sex-specific adaptations in placental development in response to maternal nutrition [31]. Similarly, human placentas exhibit sex-based differences in size and gene expression throughout gestation, potentially contributing to distinct intrauterine growth patterns [32, 33]. Recent research has highlighted sexual dimorphism in placental mitochondrial respiration, particularly in the utilization of glutamine, fatty acids, and glucose. Notably, placentas from obese women with gestational diabetes mellitus showed increased reliance on glucose and fatty acids for baseline respiration and reduced metabolic flexibility in utilizing these substrates, exclusively in male fetuses [34]. These findings underscore the importance of considering fetal sex in understanding the complex interplay between maternal health, placental function, and fetal development [34].

The intricate interplay between placental fatty acid metabolism, oxidation capacity, and their impact on transport to the fetus remains poorly characterized, particularly in PE pregnancies. We postulate that PE differentially affects placental lipid metabolism, fatty acid oxidation, and transport mechanisms based on fetal sex. To investigate this hypothesis, we conducted a comprehensive analysis of term placentas from both male and female offspring of PE and normotensive pregnancies. This analysis encompassed lipid quantification and gene expression profiling of enzymes and transporters involved in fatty acid oxidation, esterification, and lipid transport.

## Methods

### Placenta Sample Collection

All procedures were conducted in accordance with the Declaration of Helsinki. Pregnant women were enrolled to donate their placenta under a protocol approved by the Institutional Review Board at the University of Wisconsin, Madison (Protocol # 2018-006). All participants gave informed written consent for sample collection and the use of their protected health information. PE was defined according to standard American College of Obstetricians and Gynecologists criteria [35]. All PE patients included in this study had late-onset mild PE without major fetal morbidity. To avoid the stress of labor, only c-section-delivered placentas were used. Placental tissue was collected from the maternal face of the placenta, avoiding under-perfused or calcified cotyledons. Several full-depth samples were collected randomly across the surface of the placenta from multiple cotyledons. Chorionic membranes and maternal decidua layers were removed, and large villous samples were further dissected into small pieces that were blotted for removal of blood and separately snap frozen in liquid nitrogen within 5 minutes of biopsy.

### Placental lipid analysis

#### Lipid extraction

Frozen placental tissues (20-30 mg) were used for the extraction and spiked with 10 μL of an internal standard and calibration mixture consisting of 500 picomoles/μL of di-myristoyl phospholipids (phosphatidyl glycerol, phosphatidyl ethanolamine, phosphatidyl serine, phosphatidic acid), phosphatidylcholine (46:0), sphingomyelin (30:1), ceramide (30:1) and triacylglycerol (15:0). To each sample, 300 μL of −20 °C chilled 75% methanol containing 1 mM butylated hydroxytoluene (BHT) was added and samples were homogenized in a Fisher Scientific bead mill using ceramic beads. Then, one mL of MTBE was added to each sample, and samples were then vortexed for 60 minutes at room temperature. 250 μL of water and 75 μL of methanol were added, and the samples were vortexed for an additional 15 minutes and then centrifuged for 15 minutes. The supernatants were collected in new test tubes, and precipitated proteins were re-extracted as above. Pooled extracts were dried overnight in a speed vac and resuspended in 500 μL of isopropanol containing 0.01% BHT.

#### Mass Spectrometry

Immediately prior to analysis, aliquots of each lipid extract were diluted in isopropanol: methanol (2:1, v: v) containing 20 mM ammonium formate. Full scan MS spectra at 100,000 resolutions (defined at m/z 400) were collected on an LTQ-Orbitrap Velos mass spectrometer (Thermo Scientific, Waltham, MA) in both negative and positive ionization modes. Scans were collected from m/z 200 to m/z 1200. For each analysis, 10 μL of sample was directly introduced by flow injection (no LC column) at 10 μL/min using an electrospray ionization source equipped with a heated ESI needle. A Shimadzu Prominance HPLC (Shimadzu Corporation, Carlsbad, CA) served as the sample delivery unit. The sample and injection solvent were 2:1 (v: v) isopropanol: methanol containing 20 mM ammonium formate. The spray voltage was 4.5 kV, ion transfer tube temperature was 275 °C, the S-lens value was 50 percent, and the ion trap fill time was 100 ms. The autosampler was set to 15 °C. After two minutes of MS signal averaging, the LC tubing, autosampler, and ESI source were flushed with 1 mL of isopropanol prior to injection of the next sample. Samples were analyzed in random order, interspersed by blank injections. Following MS data acquisition, offline mass recalibration was performed with the "Recalibrate Offline" tool in Thermo Xcalibur software (Waltham, MA) according to the vendor's instructions, using the theoretical computed masses for the internal calibration standards and several common endogenous mammalian lipid species. MS/MS confirmation and structural analysis of lipid species identified by database searching were performed using higher-energy collisional dissociation (HCD) MS/MS at 60,000 resolution and a normalized collision energy of 25 for positive ion mode and 60 for negative ion mode. MS/MS scans were triggered by inclusion lists generated separately for positive and negative ionization modes.

#### Lipid Peak Finding, Identification, and Quantitation

Lipids were identified using the Lipid Mass Spectrum Analysis (LIMSA) v.1.0 software linear fit algorithm for automated peak finding and correction of ^13^C isotope effects. Peak areas of found peaks were quantified by normalization against an internal standard of a similar lipid class. The top ~300 most abundant peaks in both negative and positive ionization modes were then chosen for MS/MS inclusion lists and imported into Xcalibur software (Waltham, MA, USA) for structural analysis on a pooled sample as described above.

### Placental gene expression analysis by quantitative polymerase chain reaction

Total RNA was obtained following homogenization of ~40 mg placental tissue in TRIzol reagent (Invitrogen, Waltham, MA), followed by cleanup using the RNeasy mini kit (QIAGEN, Valencia, CA) following the manufacturer's instructions. The integrity and concentrations of RNA were determined with DS-11 spectrophotometer (DeNovix, Wilmington, DE). Synthesis of cDNA was done with 1 μg of RNA with an iScript cDNA synthesis kit (Bio-Rad, Hercules, CA). After dilution, cDNA corresponding to 20 ng of RNA was amplified by using a CFX96 real-time thermal cycler (Bio-Rad), and a SsoAdvanced Universal SYBR Green Supermix (Bio-Rad). Primer sequences are shown in Table 1. The 2^−ΔΔCT^ method was used for analysis, and data was expressed as the fold change of the gene of interest in experimental versus control samples. All reactions were performed in duplicate, with GAPDH used as an internal control.

### Statistical Analyses

Data are presented as mean ± standard error of the mean (SEM). Statistical analysis was performed using GraphPad Prism (GraphPad Software, Boston, MA). Comparisons between PE and control groups within each fetal sex were conducted using Student's t-test, following confirmation of normality assumptions by the Shapiro-Wilk test. Statistical significance was defined as p < 0.05.

## Results

### Maternal Characteristics

Table 2 depicts maternal demographics in the study population. There are no significant differences in maternal body mass index (BMI) or maternal age between normotensive control and PE groups in both male and female fetuses. However, gestational age is significantly lower in PE groups compared to control groups for both male and female fetuses. As expected, systolic and diastolic blood pressure are significantly higher in PE groups compared to normotensive control groups for both male and female fetuses. Fetal weight does not differ significantly between normotensive control and PE groups in either male or female fetuses. The PE groups show a protein/creatinine ratio greater than 0.3. All patients are Caucasians with no current or past history of other major complications.

### Placental Lipid Quantification

Analysis of placental lipid levels in PE and control pregnancies revealed significant differences based on fetal sex. In male PE placentas, total triacylglycerol, cholesteryl esters, and free cholesterol are significantly higher compared to male control placentas (Table 3). Female PE placentas only showed a significant increase in free cholesterol compared to female control placentas, with no significant changes in total triacylglycerol and cholesteryl esters (Table 3). Additionally, phosphatidylcholine, sphingomyelin, phospholipids, and ceramides are significantly lower in male PE placentas compared to male control placentas, while no differences were observed in female placentas (Table 3).

### Placental Fatty Acid Transport Gene Expression

**Figure 1:**
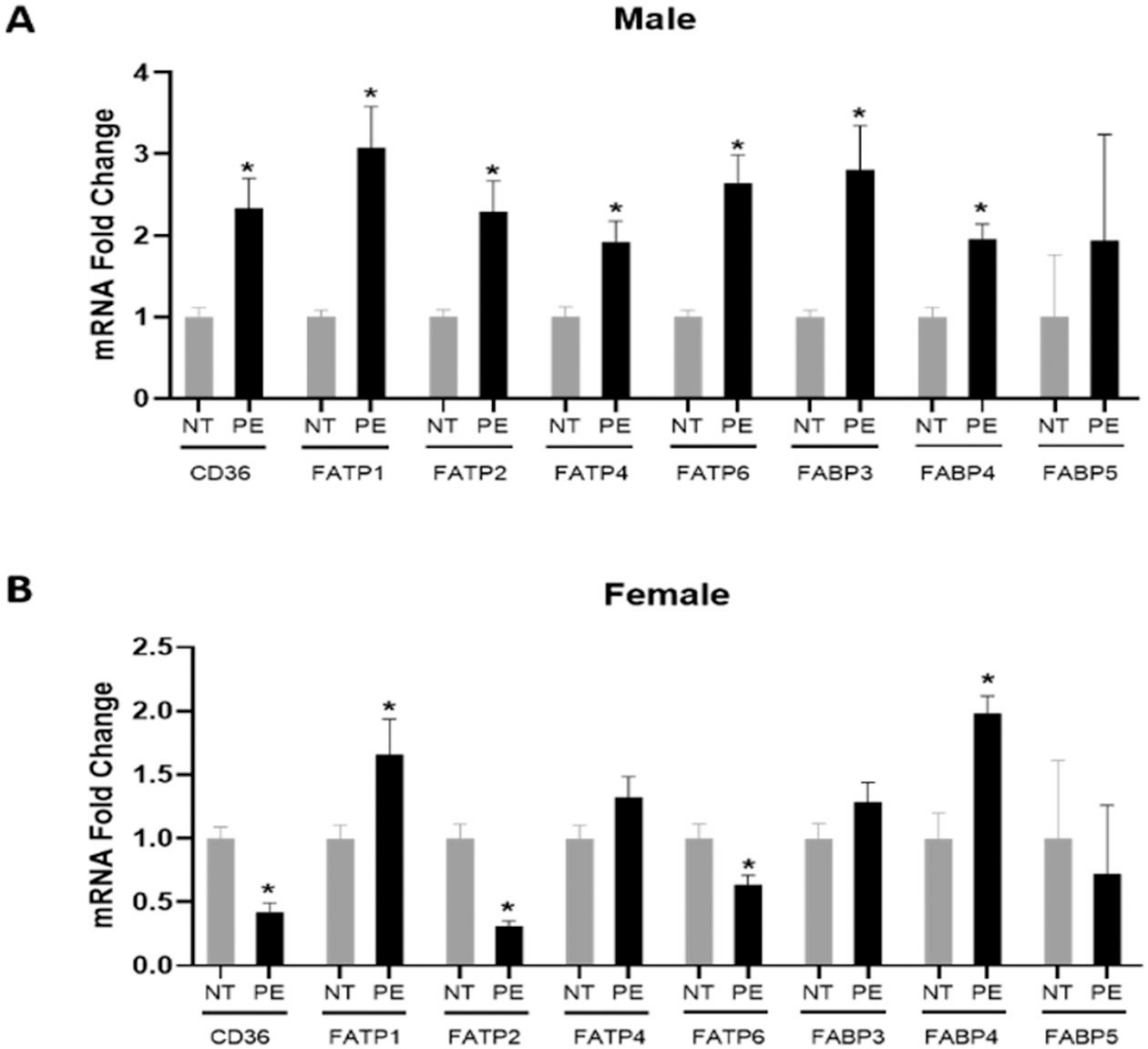
Effect of preeclampsia on placental fatty acid uptake and transport in (A) male and (B) female placentas. mRNA expression of placental genes involved in fatty acid uptake and transport was assessed in 20 placentas in each sex in normotensive (NT) control and preeclampsia (PE) groups. Data (means ± SEM) are presented as the ratio of the target gene to the reference gene GAPDH. *p<0.05 compared to respective NT controls.

In male PE placentas, there is a significant increase in the mRNA expression of CD36, FATP1, FATP2, FATP4, FATP6, FABP3, and FABP4 compared to male control placentas. In contrast, the mRNA expression of FABP5 does not significantly differ between male PE and control placentas (Figure 1A). In female PE placentas, there is a significant increase in FATP1 and FABP4 mRNA expression compared to female control placentas. However, a significant decrease was observed in CD36, FATP2, and FATP6 mRNA expression in female PE placentas compared to female control placentas. The mRNA expression of FABP3 and FABP5 does not differ significantly between female PE and control placentas (Figure 1B).

### Placental Lipid Esterification Gene Expression

**Figure 2:**
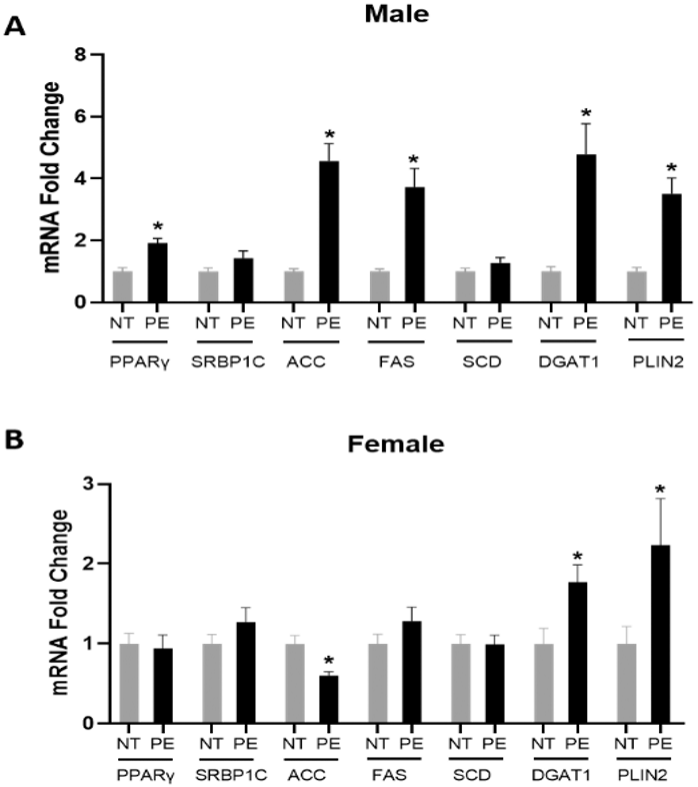
Effect of preeclampsia on the placental lipid esterification pathway in (A) male and (B) female placentas. mRNA expression of placental genes involved in lipid esterification was assessed in 20 placentas in each sex in normotensive (NT) control and preeclampsia (PE) groups. Data (means ± SEM) are presented as the ratio of the target gene to the reference gene GAPDH. *p<0.05 compared to respective NT controls.

In male PE placentas, there is a significant increase in the mRNA expression of PPARγ, ACC, FAS, DGAT1, and PLIN2 compared to male control placentas. In contrast, the mRNA expression of SRBP1c and SCD does not significantly differ between male PE and control placentas (Figure 2A). In female PE placentas, there is only a significant increase in DGAT1 and PLIN2 mRNA expression compared to female control placentas. However, a significant decrease is observed in ACC mRNA expression in female PE placentas compared to female control placentas. The mRNA expression of PPARγ, SRBP1c, FAS, and SCD does not differ significantly between female PE and control placentas (Figure 2B).

### Placental Fatty Acid Oxidation Gene Expression

**Figure 3:**
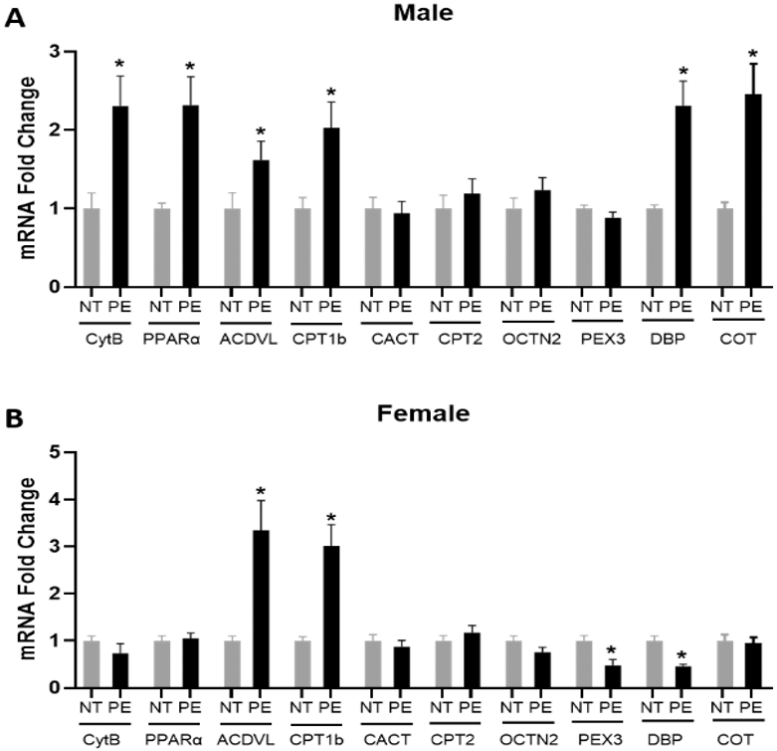
Effect of preeclampsia on the placental fatty acid oxidation pathway in (A) male and (B) female placentas. mRNA expression of placental genes involved in fatty acid oxidation was assessed in 20 placentas in each sex in normotensive (NT) control and preeclampsia (PE) groups. Data (means ± SEM) are presented as the ratio of the target gene to the reference gene GAPDH. Mitochondrial number was estimated by the ratio of CytB/β-actin DNA. *p<0.05 compared to respective NT controls.

In male PE placentas, there is a significant increase in the mRNA expression of Cytb, PPARα, ACDVL, CPT1b, DBP and COT compared to male control placentas. In contrast, the mRNA expression of CACT, CPT2, OCTN2 and PEX3 does not significantly differ between male PE and control placentas (Figure 3A). In female PE placentas, there is only a significant increase in the mRNA expression of ACDVL and CPT1b compared to female control placentas. However, a significant decrease was observed in PEX3 and DBP mRNA expression in female PE placentas compared to female control placentas. The mRNA expression of Cytb, PPARα, CACT, CPT2, OCTN2, and COT does not differ significantly between female PE and control placentas (Figure 3B).

## Discussion

Placental lipid metabolism is essential for maintaining proper placental function, ensuring positive pregnancy outcomes, and supporting healthy fetal growth [30, 36]. To our knowledge, this is the first comprehensive investigation of the transcriptomic changes in enzymes involved in fatty acid oxidation and esterification, lipid transporters, and lipid content within placental tissue in the context of PE. Our study reveals a striking sexual dimorphism in the impact of PE on placental lipid metabolism. Specifically, we observed elevated levels of placental triacylglycerol and cholesteryl esters in pregnancies with male fetuses, accompanied by increased expression of genes involved in fatty acid esterification, β-oxidation, and transport. In contrast, pregnancies with female fetuses did not exhibit significant alterations in triacylglycerol and cholesteryl ester levels, and fewer lipid metabolism genes were affected.

Consistent with previous studies [8, 37, 38], our data demonstrates elevated placental lipid content in PE pregnancies compared to controls. Notably, we observed a sex-specific effect, with male PE placentas exhibiting a more pronounced lipid accumulation than female PE placentas. Specifically, triacylglycerols, cholesteryl esters, and free cholesterol were significantly higher in male PE placentas compared to their respective controls. In contrast, only free cholesterol was significantly elevated in female PE placentas compared to controls. Previous studies have also reported increased placental lipid content in PE, although fetal sex was not considered [8]. The abundance of transport proteins at the maternal-placental interface highlights its importance in regulating fatty acid uptake into the placenta. Several membrane transporters, including CD36 (fatty acid translocase) and FATPs (fatty acid transport proteins 1-6), are implicated in placental fatty acid uptake [39]. Once within the trophoblast, fatty acid-binding proteins (FABPs) facilitate intracellular transport to mitochondria for oxidation, to organelles for esterification and storage, or to the fetal-facing trophoblast membrane for fetal delivery [40]. While our data shows upregulation of fatty acid uptake gene expression (CD36, FATP1, FATP2, FATP4, FATP6) in male PE placentas, this was not observed in female PE placentas, which instead showed downregulation of CD36, FATP2, and FATP6 and upregulation of only FATP1. Previous reports [38, 41], without consideration of fetal sex, have shown decreased CD36 and increased FATP1 mRNA expression in term PE placentas. However, we observed upregulation of major intracellular fatty acid carriers FABP3 and FABP4 in male PE placentas and FABP4 in female PE placentas. Consistently, Yan et al. reported that placental FABP4 mRNA expression is higher in placentas of PE women compared to controls, but the sex of the placenta is not reported [42]. Our findings suggest increased placental uptake in male, but not female, PE placentas. However, the upregulation of intracellular fatty acid carriers FABP3 and FABP4 in male PE placentas and FABP4 in female PE placentas indicates augmented intracellular fatty acid trafficking in both sexes in PE pregnancies.

Our data supports the notion that the upregulation of key proteins in the lipid esterification pathway may direct fatty acids toward storage in PE placentas. This is particularly evident in male PE placentas, where we observed a significant increase in the expression of PPARγ, a master regulator of lipid esterification and storage [43-45]. This upregulation of PPARγ could be a response to elevated placental fatty acid concentrations, potentially stemming from increased placental uptake [46]. Although this mechanism requires further investigation in placental tissue, similar findings have been reported in adipose and hepatic tissues of mice and non-pregnant individuals [43, 45]. Notably, in a mouse model, rosiglitazone-induced placental PPARγ activation increased placental fatty acid uptake but decreased transfer to the fetus due to enhanced lipid storage [43]. PPARγ is known to stimulate the transcription of ACC, FAS, DGAT1, and other key genes involved in lipid esterification and storage [44, 47, 48]. Consistently, the increased expression of PPARγ in male PE placentas was accompanied by a significant upregulation of ACC and FAS, key enzymes involved in de novo lipogenesis [49]. This suggests that increased fatty acid uptake may lead to enhanced fatty acid synthesis in male PE placentas. Moreover, the upregulation of DGAT1 and PLIN2, genes involved in triacylglycerol synthesis [48] and lipid droplet formation [50], respectively, further supports the idea of increased lipid storage in these placentas. In contrast, female PE placentas did not exhibit such a pronounced upregulation of lipid esterification genes. While DGAT1 and PLIN2 were upregulated, ACC was downregulated, suggesting a less active de novo lipogenesis pathway compared to male PE placentas. This observation aligns with the lack of significant changes in triacylglycerol and cholesterol ester levels in female PE placentas. These findings highlight a sex-specific effect of PE on placental lipid metabolism, with male placentas exhibiting a greater propensity for lipid accumulation through enhanced fatty acid uptake, synthesis, and storage. The upregulation of PPARγ in male PE placentas may be a key driver of this process, and further investigation into the mechanisms underlying this phenomenon is warranted.

In addition to influencing tissue fatty acid content and availability for esterification and storage, placental fatty acid oxidation provides ATP essential for critical functions such as hormone synthesis, which regulates maternal adaptations to pregnancy and parturition [51-53]. In male PE placentas, there is a significant upregulation of key genes involved in mitochondrial fatty acid oxidation, including CPT1b (the rate-limiting enzyme) [54, 55], its positive regulator PPARα [56], and ACDVL, which catalyzes the first step of mitochondrial fatty acid β-oxidation. This is accompanied by an increase in Cytb, a marker of mitochondrial number, suggesting a potential compensatory mechanism to enhance overall oxidation capacity in response to the increased lipid load observed in PE. Furthermore, in male PE placentas, we observe upregulation of DBP and COT, key components of the peroxisomal β-oxidation machinery. This could indicate an increased reliance on peroxisomal fatty acid oxidation in response to the excess fatty acid accumulation in PE, as peroxisomes can oxidize shorter fatty acids that bypass the CPT1b to enter mitochondria through diffusion to maintain overall β-oxidation capacity [29, 57, 58]. However, this compensatory mechanism may have detrimental effects due to the generation of hydrogen peroxide, a reactive oxygen species that can damage mitochondria if not adequately scavenged by antioxidants [59, 60]. Interestingly, female PE placentas show a different response, with only CPT1b and ACDVL being upregulated. The downregulation of PEX3, a regulator of peroxisome biogenesis and integrity, and DBP, suggests a suppression of peroxisomal fatty acid oxidation in female PE placentas. This sexually dimorphic response in peroxisomal activity may contribute to the differential susceptibility of male and female fetuses to the adverse effects of PE. These findings align with previous studies reporting increased expression of fatty acid oxidation-related genes in PE animal models and PE [61, 62], although other studies have shown decreased expression [38, 63]. Our findings highlight the complex and sexually dimorphic regulation of placental fatty acid oxidation in PE. The upregulation of mitochondrial and peroxisomal oxidation pathways in male placentas, coupled with the increase in mitochondrial number, suggests a compensatory mechanism to handle excess fatty acids, albeit with potentially negative consequences due to increased oxidative stress. In contrast, the suppression of peroxisomal oxidation in female placentas may reflect a different adaptive strategy to PE.

**Figure 4:**
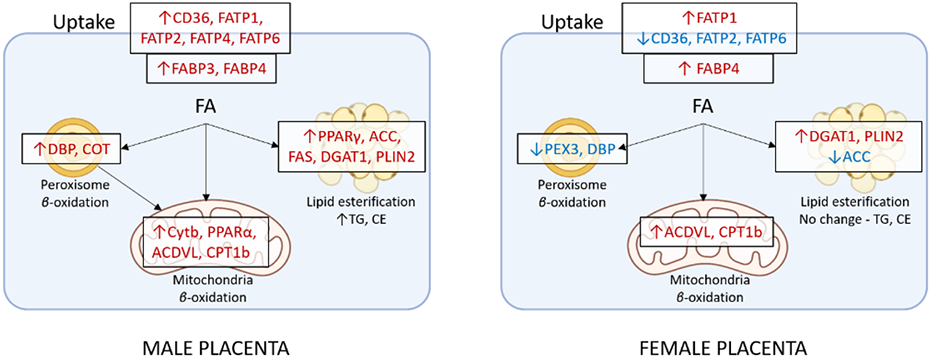
Schematic summarizing sexually dimorphic alterations in placental lipid metabolism in PE. In male placentas, preeclampsia (PE) upregulates fatty acid (FA) transport, β-oxidation, and esterification, leading to increased triacylglycerol (TG) and cholesterol ester (CE) accumulation. In contrast, female placentas exhibit a more complex response, with altered expression of fatty acid transport and oxidation genes, but no significant changes in TG and CE levels. These findings suggest that male placentas may be more vulnerable to the lipotoxic effects of PE. Red arrows – Upregulated; Blue arrows – Downregulated.

In conclusion, our study demonstrates a marked sexual dimorphism in placental lipid metabolism in PE, with male fetuses exhibiting greater susceptibility to dysregulated fatty acid oxidation, esterification, and transport compared to female fetuses (Figure 4). These findings underscore the importance of considering fetal sex in future research on PE, as it may reveal novel insights into the underlying mechanisms and potential therapeutic targets. However, it is important to note that our study is limited to the analysis of term placentas, and further prospective studies are needed to investigate protein expression and activity of enzymes/transporters and the developmental trajectory of these alterations throughout gestation. Additionally, future studies should also assess lipid levels in maternal and fetal tissues or plasma to gain a more comprehensive understanding of the interplay between placental lipid metabolism, fetal development, and long-term health outcomes. Ultimately, this line of research may contribute to the development of personalized interventions for PE based on fetal sex and contribute to a deeper understanding of the placenta's role in shaping offspring health.

## Figures and Tables

**Table 1.** Quantitative real-time PCR primer sequence.

Gene Name	Forward Primer	Reverse Primer
CD36	CAGGTCAACCTATTGGTCAAGCC	GCCTTCTCATCACCAATGGTCC
FATP1	TGACAGTCGTCCTCCGCAAGAA	CTTCAGCAGGTAGCGGCAGATC
FATP2	GTGGAGAAAGATGAACCTGTCCG	CTGAGCCTTTGCTCCAGCATAG
FATP4	GGACCAACTTTTCCAGCCGCTT	TGCGGCTATTGAAACCACAGGC
FATP6	AGCCACCATGTTGTCTCACTCC	ACCAAAAGCCCACAGGACAGCA
FABP3	GTGGAGTTCGATGAGACAACAGC	TGGTCTCTTGCCCGTCCCATTT
FABP4	ACGAGAGGATGATAAACTGGTGG	GCGAACTTCAGTCCAGGTCAAC
FABP5	GGTGCATTGGTTCAGCATCAGG	TCATAGATCCGAGTACAGGTGAC
PPARγ	TCTAAAGAGCCTGCGAAAGC	GCTTGTAGCAGGTTGTCTTG
SRBP1C	GTTGGTGCTCGTCTCCTTGG	GCAGGTGACGGATGAGGTTC
ACCa	GATGTGAGCCTGCGGAATAG	AACTGCTGCCATCATAGGAC
FAS	AGATGGCTTGCTGGAGAACC	AGTTGCTCTGTCCCGCATTG
SCD	GGTTTCACTTGGAGGTGTGG	TTGATGTGCCAGCGGTACT
DGAT1	TCCTTGAGATGCTGTTCTTC	CCAGTAGAAGAAGATGAGCC
PLIN2	GATGGCAGAGAACGGTGTGAAG	CAGGCATAGGTATTGGCAACTGC
CytB	CCAACATCTCCGCATGATGAAAC	TGAGTAGCCTCCTCAGATTC
PPARα	TTTCTGTCGGGATGTCACAC	TCTTCAAGTAGGCCTCGTAG
ACDVL	TTGGCAGTGCTCTAAAGAAT	GCAGCAGAAACTGTTCATTG
CPT1b	CGTTCCTGTACCACGAGTC	GAAGAGGTGCCTGTCGATCC
CACT	TCCCAGCTAGTGGAATGTAT	CCCTTTGTACAAGGATGTGA
CPT2	AAACCCTCACTATTGACTGC	CACTCAACCATCATCTGCT
OCTN2	GACCATATCAGTGGGCTATTT	CTGCATGAAGAGAAGGACAC
PEX3	ACCAGAGGACTTGCAATATG	TACAGCCACAGTACTTCTTG
DBP	ATTGTAACACCATTGCTCCT	CCCAGCGTAATTTTCCAATC
COT	TCACCCGGATACGTTTATTC	CGATCAAATCCTTTTCCAGC
GAPDH	GTCTCCTCTGACTTCAACAGCG	ACCACCCTGTTGCTGTAGCCAA

**Table 2:** Maternal demographics in the study population

Population characteristics	Male			Female		
	Control	PE	p-value	Control	PE	p-value
**Maternal BMI**	24.1 ± 1.6	25.8 ± 1.3	NS	23.9 ± 0.7	24.9 ± 1.3	NS
**Maternal age (years)**	30.2 ± 1.0	28.9 ± 1.4	NS	33.7 ± 2.2	31.9 ± 2.1	NS
**Gestational age (weeks)**	39.3 ± 0.6	37.2 ± 0.2	< 0.05	39.4 ± 0.3	37.4 ± 0.8	< 0.05
**Fetal Weight (grams)**	3401 ± 121	2910.3 ± 161	NS	3473.5 ± 107.1	2831 ± 248.1	NS
**Systolic BP (mmHg)**	112.1 ± 3.2	141 ± 1.2	< 0.05	109.2 ± 2.1	150.4 ± 4.1	< 0.05
**Diastolic BP (mmHg)**	75.6 ± 3.4	95.4 ± 2.3	< 0.05	70.0 ± 3.2	90.8 ± 5.1	< 0.05
**Protein/creatinine ratio (mg/ml)**	NA	> 0.3		NA	> 0.3	

PE – preeclampsia; BMI – body mass index; BP- blood pressure; n=20 for each group.

**Table 3:** Quantitative comparison of class total lipid profiles in placenta (ng/mg tissue).

Lipid Class	Male			Female		
	Control	PE	p-value	Control	PE	p-value
**Triacylglycerol**	210.85 ± 23.97	526.99 ± 109.95	< 0.05	210.08 ± 18.84	437.19 ± 163.39	NS
**Cholesteryl Esters**	71.57 ± 13.05	164.17 ± 34.05	< 0.05	52.26 ± 5.56	125.54 ± 31.30	NS
**Free Cholesterol**	8.69 ± 0.73	17.80 ± 2.24	< 0.05	7.37 ± 0.35	17.13 ± 4.96	< 0.05
**Phosphatidylcholine**	4333.82 ± 325.47	2002.37 ± 337.05	< 0.05	3749.67 ± 523.17	3251.36 ± 200.18	NS
**Phosphatidylethanolamine**	3504.87 ± 310.28	2303.73 ± 709.90	NS	3091.43 ± 440.05	4230.95 ± 166.27	NS
**Phosphatidylserine**	101.82 ± 14.08	84.25 ± 14.11	NS	92.28 ± 20.71	114.75 ± 11.22	NS
**Sphingomyelin**	3779 ± 98.97	2092.61 ± 333.61	< 0.05	3081.58 ± 247.76	3125.12 ± 264.54	NS
**Phospholipids**	8148.01 ± 658.75	4488.99 ± 1048.66	< 0.05	7136.89 ± 982.28	7806.97 ± 371.15	NS
**Ceramide**	82.15 ± 9.42	35.31 ± 8.57	< 0.05	67.71 ± 6.60	73.89 ± 6.61	NS

PE – preeclampsia; n=20 for each group.

## Data Availability

All data are incorporated into the article and are available upon request.
